# COVID-19 Burden in Long-Term Care Facilities in the Omicron Era: Public Health Action Not Yet Redundant

**DOI:** 10.3390/v15030752

**Published:** 2023-03-14

**Authors:** Dimitra Krystallaki, Christina-Anna Kavakioti, Maria Gkova, Soultana Sypsa, Kyriaki Tryfinopoulou, Aikaterini Gavrili, Aikaterini Dimitriou, Spyridon Sapounas, Dimitrios Paraskevis, Kassiani Mellou

**Affiliations:** Directorate of Epidemiological Surveillance and Intervention for Infectious Diseases, National Public Health Organization (EODY), 11523 Athens, Greece

**Keywords:** SARS-CoV-2, COVID-19, Omicron, BA.1, BA.2, BA.5, long-term care facilities, LTCF, public health, infection prevention and control, IPC

## Abstract

Since the beginning of the pandemic, public health authorities have provided support to long-term care facilities (LTCFs) for the implementation of risk mitigation measures. Nevertheless, the necessity of these measures has been doubted, especially after vaccines and antiviral treatment became available. Here, we present the burden of COVID-19 infection in LTCFs during the first 9 months of 2022 across Greece. We tested the possible association of LTCF characteristics and public health response with the occurrence of clusters (two or more linked cases in LTCFs) with facilities recording one case as reference. After excluding LTCFs with sporadic cases, we tested the effect of the abovementioned variables on attack rate (cases/total number of persons in the LTCF). The disease burden in LTCFs was high and substantially varied among facilities, with hospitalization and case fatality rates ranging from 2 to 80% (median 14%, IQR 27%) and from 1 to 50% (median 5%, IQR 7%), respectively. The probability of transmission inside the facility increased when notification of public health authorities was delayed (*p*-Value < 0.001) after adjusting for vaccination status and phase of the pandemic. Results showed that active support from public health authorities is still important in reducing the burden in LTCFs.

## 1. Introduction

Long-term care facilities (LTCFs) for the elderly and the chronically ill pose a high risk of transmission of respiratory pathogens among residents and staff, with SARS-CoV-2 being the most recent concern [1]. Residents are also at increased risk for severe illness and associated complications as they are usually frail with comorbidities [2].

The World Health Organization (WHO) declared COVID-19 a pandemic on 11 March 2020. As of February 2023, more than 673 million cases and 6.85 million deaths were recorded worldwide [3,4]. From the onset of the pandemic, LTCFs have been substantially affected [5,6]. These settings marked a high burden and associated mortality across waves, especially before the advent of vaccination [5]. Thus, they were prioritized in vaccination campaigns and were the focus of preventive and risk mitigation strategies in many countries [7].

In Greece, a vigorous nationwide vaccination program was implemented by the Greek National Public Health Organization (EODY) as soon as the vaccine became available; most of the facilities had completed a two-dose regimen by spring of 2021, and the third dose was offered in autumn of 2021 after the surge in cases caused by the Delta variant. The Omicron variant, first identified on 29 November 2021 in the country, quickly became dominant, and by the beginning of 2022, SARS-CoV-2 infections were again on the rise even among fully vaccinated persons. The fourth dose was first administered in May 2022.

EODY is also the competent authority that coordinates response in the event of cases in a LTCF. A specially trained team of healthcare professionals performs risk assessment and provides infection control guidance to the LTCF via communication with the scientific director or another COVID-19 appointed person in the facility. A standard response protocol is followed.

The facility is set under surveillance for at least 10 days following the confirmation of a new case (day 0). The team guides the use of non-pharmaceutical interventions, such as physical distancing, appropriate use of personal protective equipment, case isolation, and contact tracing and testing of all residents and staff members every 4 days starting at day 0. The physician of the LTCF is also informed on available antiviral treatment options and the facility is monitored for the occurrence of new cases. If no new cases occur for at least 10 days of periodic testing and distancing measures, the monitoring period is over.

More than 2 years into the pandemic, several questions have been raised on the effectiveness of preventive measures —mandatory at times— and their necessity, given the psychological impact of isolation among other risks [8,9]. This questioning attitude has come forward more intensively now, considering the relatively milder disease caused by Omicron, the high vaccination coverage of the population, and the availability of disease-specific antivirals [10,11,12].

In post-pandemic circumstances, whether advances in combatting the virus can adequately downgrade infection control and public health interventions is not clarified yet. Thus, we aimed to assess the need for keeping the enhanced response protocol implemented by EODY and identify high risk conditions and modifiable practices for adjusting this response.

In this study, we first sought to estimate the burden of COVID-19 in people living in LTCFs in relation to community burden in the first 9 months of 2022 across Greece during the Omicron dominance period. Subsequently, we explored which factors were associated with further transmission inside the facility after the occurrence of the first case and which factors were associated with attack rate in case of clusters.

## 2. Materials and Methods

### 2.1. Study Population

The enrollment started on 3 January 2022 (ISO week 01/2022), and the last follow-up was completed on 9 October 2022 (40/2022). LTCFs from all regions of Greece were included.

The criteria for inclusion of a facility in the study were met when (i) the hosted population was relatively stable (closed setting); (ii) residents were over 65 years of age; and (iii) at least one confirmed SARS-CoV-2 case was detected among residents or staff (including LTCF associated cases, i.e., residents testing positive early in hospital admission with no other known exposure).

Exclusion criteria were met if the case had no physical presence in the premises of the facility during the transmission period, i.e., 3 days prior to symptom onset or first positive test, whichever occurred earlier.

The eligible LTCFs were then allocated to two groups based on the number of reported cases. The first group included facilities with a cluster of two or more epidemiologically linked cases, i.e., confirmed cases that occurred in the same LTCF within 10 days and had known physical presence within two meters and the comparison group included facilities with a single confirmed case, i.e., a minimum of 10-day interval from any previously reported case in the same facility.

No distinction based on the type of care provided in the LTCF was made as LTCFs for the elderly in Greece are mostly of the mixed type.

The capital region of Attica contributes 36% of the country’s population. Therefore, in the analysis, the variable location included two groups: facilities situated in the capital region and facilities in all other regions [13].

### 2.2. Data Sources

When not under monitoring, LTCF testing strategies included mandatory weekly screening for personnel, optional periodic screening for residents, and symptomatic testing for both groups.

In Greece, all SARS-CoV-2 cases confirmed by a molecular test (RT-PCR) or rapid antigen test (RAT) are required to be recorded to the national COVID-19 registry. Healthcare professionals that perform SARS-CoV-2 tests, including LTCF staff, have access to the registry, and along with the result, they record basic epidemiological information.

EODY uses the digital registry as the main source of information on the daily occurrence of new LTCF cases. Other sources of information are direct communication from an LTCF staff member or other local public health authority and review of media articles.

Data on the characteristics of the facilities and cases were collected by contacting the COVID-19 appointed person of the LTCF initially on the occurrence of the first detected case and at least once by the end of follow-up. The relevant data were provided by regularly updating a specially designed reporting form. Each LTCF received a unique depersonalized code, and data were recorded in a database created for this purpose.

Data on the weekly testing rate were retrieved from the database of EODY and data on variants circulation from the country-wide genomic surveillance network. Randomly selected community PCR samples and samples collected in a targeted way were routinely sequenced in the public health laboratories during the study period.

### 2.3. Vaccination Status of the LTCF

For categorizing the facilities regarding their vaccination status, we recorded the date that at least 80% of LTCF persons received each dose (typically on the first visit of the EODY team) [1]. The vaccination status of the facility was determined based on the date of the last dose, given that it was at least 14 days before the first notified case in the LTCF. If the case was within the 14-day interval, the previous dose of the vaccine was taken into account [14].

LTCFs were categorized in three groups: those vaccinated with the third dose of the vaccine more than 5 months prior, those vaccinated with a third dose less than 5 months prior, and those having received the fourth dose (fourth dose had not been available for more than 5 months at the time of the study) [15].

### 2.4. Statistical Analysis

Each LTCF was set as the unit of statistical analysis. We described the distribution of LTCFs reporting their first case over time in relation to the community notification rate and circulating strains.

To further assess the burden, we presented hospitalizations and deaths attributable to COVID-19 based on the judgement of the facility’s coordinating doctor and the responsible doctor in EODY among residents expressed as hospitalization and case fatality rate (number of hospitalizations or deaths per total cases, respectively) [16]. For comparison reasons, hospitalization and case fatality rate data in community dwelling adults aged over 65 years were used, accessed from the national registry.

We performed two analyses.

In the first analysis, we tested the possible association of several variables, i.e., the vaccination coverage of residents and staff, the vaccination status of the facility, the location (capital vs. all other regions), the index case attribute (resident vs. staff member), the LTCF population size (residents and staff), the delay between first case detection and EODY notification/guidance, and the staffing ratio (number of staff members per total resident count) with the occurrence of clusters of two or more epidemiologically linked cases in LTCFs (transmission inside the facility after the first identified case) using facilities that recorded a single case as reference group.

The second analysis regarded only LTCFs with clusters of cases, and LTCFs with sporadic cases were excluded. The previously mentioned variables were examined for their effect on the size of the cluster (attack rate) measured as the percentage of total cases over the total number of persons in the LTCF (staff and residents).

Numeric variables were presented as mean and standard deviation or median and interquartile range. Categorical variables were expressed as absolute (N) and relative frequency (%) in each category of the variable.

Normality was tested using the Kolmogorov–Smirnov test (when the sample size was n ≥ 30) and Shapiro–Wilk test (when the sample size was n < 30). If the assumption of normality was not met in any category of the variable, nonparametric tests were performed.

Multiple logistic regression and linear regression models were performed for both analyses.

A variable with the different infectivity periods during the study period was included in the multivariable models to adjust for community incidence and circulation of variants.

Infectivity periods were discerned as follows: A wave was defined as the period where a sustained increase in cases was observed [17,18]. The wave start date was set as the first day of the ISO week in which the notification rate increased compared to the previous week and this change was sustained for at least 2 consecutive weeks. The wave end date was defined accordingly as the last date of the week in which the notification rate decreased. A surge in cases (wave peak) is followed by periods with a low notification rate (plateau) until the next peak [17].

All the statistical tests were performed at the statistical significance level of 5%. Data were analyzed using R programming language and SPSS 23 for Windows.

### 2.5. Ethical Considerations

EODY is authorized by the Greek law to process COVID-19 epidemiological data for public health purposes. No personal data were used. The study was conducted in accordance with the national and European Union regulations and approved by the Institutional Review Board of EODY.

## 3. Results

**Figure 1:**
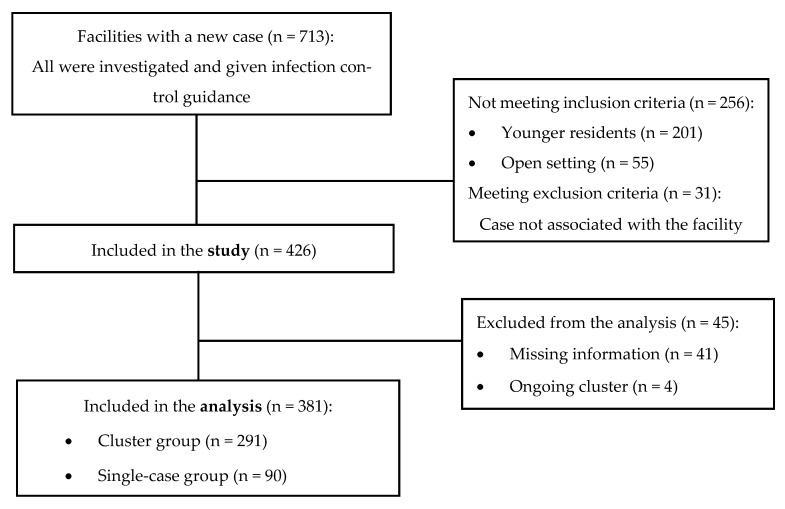
Long-term care facilities reporting a new COVID-19 case from January to September 2022 and inclusion to the study.

Of the 713 facilities with a reported case in the study period, 381 (53.4%) met study criteria (Figure 1). Of them, 59.1% were located at the capital region.

The cluster group included 291 facilities and the single-case group included 90 facilities. In the cluster group, the median number of cases among residents and staff during the monitoring period was 17 (IQR 32).

### 3.1. Notification Rate and Waves

**Figure 2:**
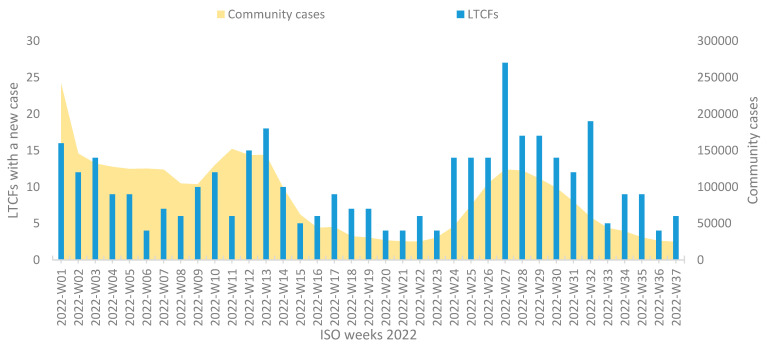
Weekly number of long-term care facilities (LTCFs) reporting SARS-CoV-2 cases by date of the first detected case and number of new community cases by date of diagnosis (per 10,000) in Greece during first 9 months of 2022. LTCFs hosting a stable population of elderly residents of 65 years or more were included.

One partial and two complete wave peaks evolved in the community during the study period. The partial corresponded to ISO week 1 (3 to 9 January 2022) and was in its downward phase. The second spanned weeks 9 to 16 (28 February to 24 April 2022). The third spanned weeks 22 to 36 (30 May to 11 September 2022). The six distinct infectivity periods are peak 1, plateau 1, peak 2, plateau 2, peak 3, and plateau 3 (Figure 2).

The average number of COVID-19 tests declined over the waves with 39,486; 35,031; and 6478 in the first, second, and third waves, respectively.

In the first Omicron wave, 16 LTCFs (4.2%) reported case/cases; in the second wave, 82 (21.5%) reported cases; and in the third wave, 185 (48.6%) reported cases. Additionally, 98 LTCFs reported the identification of a case or cases between waves. The median number of LTCFs reporting their first case was 9 per ISO week with the highest value being 27 (reported in week 27) and the lowest being 4 (in weeks 06, 20, 21, 23, 36) (Figure 2).

On average, for every 100,000 new cases in the community, 12 new LTCFs reported the occurrence of one or more cases. This ratio progressively increased over the wave peaks from 7 LTCF cases per 100,000 community cases in the first wave peak to 19 in the third one.

**Figure 3:**
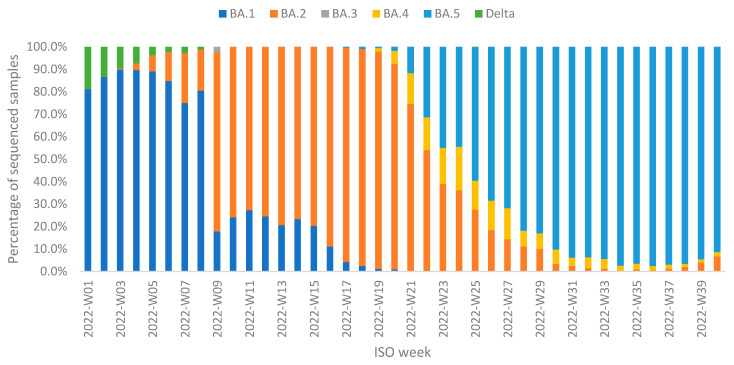
Variants and subvariants detected in sequenced community samples from ISO week 1 to 40.

Regarding variant circulation, the dominant Omicron sub-lineage (more than 80% of total samples sequenced) in the first wave was BA.1, in the second wave it was BA.2, and in the third wave BA.5. Sub-lineages BA.3 and BA.4 were detected less often (on average 2.4 and 6.9% of sequenced samples per week, respectively) (Figure 3).

### 3.2. Hospitalizations and Deaths

The hospitalization rate was 6.6% (454 hospitalizations in 6903 confirmed cases). In the sporadic case group, hospitalization data were available for 77 facilities (86%); a single case was hospitalized in 13% of the facilities. In the cluster group, hospitalization data were available for 284 of them (98%); on average, the hospitalization rate was 6.5% (444 hospitalizations in 6826 total cases). More specifically, in two-thirds of the clusters, no residents were hospitalized and for the remaining one-third, hospitalizations varied greatly, ranging from 2 to 80% of total cases (median 14%, IQR 27%). Overall, 91 facilities documented a hospitalization rate higher than the community rate of 8.2%.

The case fatality rate was 2.3% (158 attributable deaths in 6886 total cases); however, the number of deaths varied. Among the 90 facilities with a sporadic case, 77 facilities provided data (86%), and no deaths were recorded. In 291 clusters, 281 provided data (97%), with an average ratio of 2.3% (158 in 6809); in most clusters (71%), no deaths were recorded, whereas in the remaining clusters, the case fatality rate ranged from 1 to 50% (median 5%, IQR 7%). Overall, five facilities had a higher case fatality rate than the respective community rate of 2.2%.

### 3.3. Univariate and Multivariable Analysis

Descriptive characteristics of facilities and cases are summarized in Table 1, and the results of the two univariate analyses are presented in Table 2 and Table 3 for the two outcomes, the event of a cluster and the attack rate, respectively.

In the multivariable analysis, an independent association with recording a cluster instead of a sporadic case was found with two factors: the index case being a resident and time of infection control guidance given by EODY (*p* = 0.003 and *p* < 0.001, respectively; see Table 4). A resident presenting as the first case instead of staff was 2.76 times (95% CI 1.44, 5.46) more likely to lead to a cluster in the facility. A delay in receiving guidance by EODY of 2.4 days (1.6, 3.3) was recorded in facilities with clusters compared to facilities with a single case.

As shown in Table 5 in the multivariable analysis, facilities with smaller hosted populations were independently associated with higher attack rates.

## 4. Discussion

In the Omicron dominance period, notification of cases in the population of LTCFs was an everyday fact; however, the daily number of new LTCFs reporting COVID-19 cases fluctuated greatly through time, grossly reflecting the circulation of the virus in the community. Increases and decreases in new LTCFs with cases followed the peaks and valleys of infectivity waves in the community, depicting that these closed settings are not impervious to virus entry despite extensive mandatory preventive measures in place [18].

In the last wave, new LTCF cases seemed to increase disproportionately compared to the community notification rate. However, the total testing rate was lower during this wave (81.5% reduction from the second wave). Less testing is part of a global phenomenon described by WHO as pandemic fatigue—an expected and natural response to a prolonged public health crisis [19].

LTCFs experienced a markedly different situation with regards to testing∙ while community measures were becoming less strict or others were dropped completely, LTCFs were high in national surveillance priorities, in line with ECDC guidance [20]. Legislation mandated a series of preventive measures in these settings including periodic screening, quarantine, and serial entry tests in new residents and restricted visitation policy with obligatory pre-visit testing. These measures remained effective throughout the pandemic with minimal adaptations, and thus LTCFs demonstrated a relatively stable testing rate [18]. The difference in LTCF notification rate across the waves may be the result of a disproportionally increased monitoring and testing rate in these settings compared to the general population.

In most facilities, severe cases of COVID-19 were a rare event. The hospitalization rate was lower in LTCFs compared to the community. One possible explanation is that early diagnosis in combination with guidance by EODY enabled timely initiation of antivirals and prevented progression to severe disease in these high-risk individuals. Additionally, the almost universal vaccination coverage of the LTCF population may account for that difference given the high vaccine effectiveness against severe disease [12,21]. Nevertheless, in almost one-fourth of the facilities, the hospitalization rate exceeded the rate in the community. These differences may reflect the varying circumstances among the facilities that lead to the coordinating physician’s decision of referring a resident to a secondary or tertiary healthcare center.

In this study, the aggregate case fatality rate in Greek LTCFs was 2.3%. This rate lies between the reported case fatality rate of 0.6% in US-based LTCF residents in the same year and the rate of 13% of LTCFs in European countries in 2021; however, the extent of this comparison is limited given the differences in the way these rates were measured [1,22].

Independent risk factors for the first LTCF case to result in secondary transmission and cause a cluster were the index case being a resident and the higher delay of IPC guidance by EODY. The pandemic phase during which the first case occurred and the overdue vaccination status, although statistically significant in the univariate analysis, did not reach significance after adjusting their effect with other factors.

When the first identified case was a resident, there was an almost three times higher probability to have a cluster of cases in the LTCF compared to events when the first identified case belonged to the staff. Staff members use a face mask at all times in the facility and undergo weekly testing, as is stated in the legislation [19]. On the other hand, residents usually do not use masks, they are usually tested upon symptoms, and are mostly housed in shared rooms. Consequently, a case in this group is often detected after secondary transmission has already occurred in close contact and before early protective measures are implemented [23].

Facilities that delayed the report of cases did not receive timely infection control instructions and had a higher chance of experiencing a cluster. The instructions focus on risk mitigation strategies; isolation and quarantine, increased testing, and appropriate personal protective equipment are most effective before extensive virus spread. This highlights the importance of establishing an easily reachable team with infection preventionists in local or central public health units in an effort to halt transmission in the facility already from the first case, as was evident in other studies with a similar intervention [24,25].

The only independent risk factor for a higher attack rate was the lower number of persons present in the facility. The circulating strain, the lower staffing ratio, overdue vaccination status, and a resident as index case appeared statistically significant in the univariate analysis but not after adjusting for other variables. A smaller LTCF population has been reported to be protective in a similar study in Italy [26]. In Greece, facilities with higher bed capacities and more staff tend to have more resources, employ more experienced and specialized healthcare workers and can more easily relocate and cohort cases and contacts, actions that can mitigate transmission when multiple cases are present [27,28].

### Limitations

The study had several limitations. No data on individual cases were collected, and only aggregate data on the LTCF level were available. Additionally, results cannot be generalized to facilities with younger hosted populations. When testing for risk factors, facilities with single cases were used as comparison group instead of facilities with no cases for which the respective information was not available. Severe outcome rates may have been subject to bias as case outcomes were reported by the LTCF healthcare staff.

## 5. Conclusions

Even though preventive measures in the community have been loosened or abandoned, the LTCFs and other congregate settings continue to draw increased public health interest. This study showed that there is a great variability of disease burden among different LCTFs and that the early notification of new cases by the LTCF and support from public health authorities is still important in reducing burden in the post-critical pandemic phase. Thus, it is not yet time to abandon these actions.

## Figures and Tables

**Table 1:** Descriptive characteristics of long-term care facilities (LTCFs) with one or more COVID-19 cases, reported cases, and public health response in Greece during first 9 months of 2022 (n = 381).

Factors		n/N (%) or Median (Interquartile Range) ^1^
LTCF characteristics	Number of LTCF cases	
	LTCFs with ≥ 2 cases	291/381 (76.4%)
	LTCFs with a single case	90/381 (23.6%)
	Location	
	Capital region	225/381 (59.1%)
	All other regions	156/381 (40.9%)
	LTCF population size ^2^	74 (51)
	Vaccine uptake in staff ^3^ (%)	100 (0)
	Vaccine uptake in residents ^3^ (%)	100 (0.03)
	Staffing ratio ^4^	0.48 (0.26)
COVID-19 case characteristics	Index case	
	Staff	191/346 (55.2%)
	Resident	155/346 (44.8%)
	Cluster size ^5^ (% cases per total population)	26.6 (45)
Public health measures	Days until IPC ^6^ guidance	1 (3.5)
	Facility vaccination status ^7^	
	Third dose > 5 months	163/330 (49.4%)
	Third dose ≤ 5 months	116/330 (35.2%)
	Fourth dose ≤ 5 months	51/330 (15.5%)

^1^ For categorical variables, the number in question (n) in total observations (N) and percent (%) are given, and for numeric variables the median and interquartile range are given. Unanswered questions were not considered in the analyses. ^2^ LTCF population size, i.e., total number of persons regularly present in the facility, both residents and staff. ^3^ Vaccine uptake, i.e., %persons having received the primary scheme of two or more doses. ^4^ Staffing ratio, i.e., staff members per number of residents. ^5^ LTCFs with a cluster (two or more cases). ^6^ IPC, i.e., infection prevention and control. ^7^ Vaccination status, i.e., number of LTCFs with that dose in the majority of the LTCF persons.

**Table 2:** Results of univariate analysis of factors possibly associated with recording of a cluster vs. a sporadic case in long-term care facilities (LTCFs) in Greece during first 9 months of 2022.

Factor	LTCFs with a Single Case ^1^	LTCFs with ≥ 2 Cases ^1^	OR ^2^ or Mean Difference	*p* Value ^3^
Pandemic period (proxy of variants circulation)				
Peak 1	3/90 (3.3%)	13/291 (4.5%)		0.006
Plateau 1	9/90 (10.0%)	52/291 (17.9%)		
Peak 2	10/90 (11.1%)	72/291 (24.7%)		
Plateau 2	12/90 (13.3%)	19/291 (6.5%)		
Peak 3	54/90 (60.0%)	131/291 (45.0%)		
Plateau 3	2/90 (2.2%)	4/291 (1.4%)		
Vaccine uptake in staff ^4^ (%)	98.9 (98.3, 99.2)	98.3 (97.7, 98.9)	−0.6 (−1.8, 0.7)	0.386
Vaccine uptake in residents ^4^ (%)	98.2 (97.3, 99.0)	97.9 (97.1, 98.6)	−0.3 (−1.9, 1.3)	0.723
Facility vaccination status ^5^				
Third dose > 5 months	51/80 (63.7%)	112/250 (44.8%)		<0.001
Third dose ≤ 5 months	13/80 (16.3%)	103/250 (41.2%)		
Fourth dose ≤ 5 months	16/80 (20.0%)	35/250 (14.0%)		
Location				
Capital region	54/90 (60.0%)	171/291 (58.8%)	1.05 (0.65, 1.71)	0.835
All other regions	36/90 (40.0%)	120/291 (41.2%)		
Index case				
Staff	62/85 (72.9%)	129/261 (49.4%)	2.76 (1.61, 4.72)	<0.001
Resident	23/85 (27.1%)	132/261 (50.6%)		
LTCF population size ^6^	83 (71, 95)	84 (78, 90)	1 (−12, 15)	0.829
Days until IPC guidance ^7^	1.2 (0.7, 1.7)	3.6 (2.9, 4.3)	2.4 (1.6, 3.3)	<0.001
Staffing ratio ^8^	0.56 (0.48, 0.64)	0.55 (0.51, 0.58)	−0.01 (−0.09, 0.06)	0.710

^1^ For categorical variables the number in question (n) in total observations (N) and percent (%) are given and for numeric variables the median and interquartile range are given. Unanswered questions were not considered in the analyses. ^2^ OR-odds ratio. ^3^ Statistical significance level of 5%. ^4^ Vaccine uptake, i.e., %persons having received the primary scheme of two or more doses. ^5^ Vaccination status, i.e., number of LTCFs with that dose in the majority of the LTCF persons. ^6^ LTCF population size, i.e., total number of persons regularly present in the facility, both residents and staff. ^7^ IPC, i.e., infection prevention and control. ^8^ Staffing ratio, i.e., staff members per number of residents.

**Table 3:** Results of univariate analysis of factors possibly associated with the size of the cluster (percentage of epidemiologically related COVID-19 cases in total residents and staff, attack rate) in long-term care facilities in Greece during first 9 months of 2022.

Factor	Attack Rate (95% CI) ^1^	Mean Difference or Correlation Coefficient	*p* Value ^2^
Pandemic period (proxy of variants circulation)			
Peak 1	50.7 (33.4, 68.0)		<0.001
Plateau 1	42.0 (34.3, 49.8)		
Peak 2	36.4 (30.6, 42.2)		
Plateau 2	38.1 (26.9, 49.2)		
Peak 3	23.2 (19.2, 27.2)		
Plateau 3	11.5 (−6.8, 29.8)		
Vaccine uptake in staff (%) ^3^	32.2 (29.2, 35.3)	0.068	0.260
Vaccine uptake in residents (%) ^3^	32.2 (29.1, 35.2)	−0.051	0.402
Facility vaccination status ^4^			
Third dose > 5 months	30.2 (25.6, 34.7)		<0.001
Third dose ≤ 5 months	41.0 (35.8, 46.3)		
Fourth dose ≤ 5 month	19.2 (12.2, 26.1)		
Location			
Capital region	31.1 (27.3, 34.9)		0.533
All other regions	33.0 (28.1, 38.0)	1.9 (−4.2, 8.0)	
Index case			
Staff	27.0 (22.5, 31.6)		0.034
Resident	33.8 (29.4, 38.1)	6.7 (0.5, 13.0)	
LTCF population size ^5^	31.9 (28.9, 34.9)	−0.224	<0.001
Days until IPC guidance ^6^	31.9 (28.9, 34.9)	0.031	0.658
Staffing ratio ^7^	31.9 (28.9, 34.9)	−0.142	0.016

^1^ Attack rate, i.e., the size of the cluster is the percentage of epidemiologically related COVID-19 cases in total residents and staff. Unanswered questions were not considered in the analysis. ^2^ Statistical significance level of 5%. ^3^ Vaccine uptake, i.e., %persons having received the primary scheme of two or more doses. ^4^ Vaccination status, i.e., number of LTCFs with that dose in the majority of the LTCF persons. ^5^ LTCF population size, i.e., total number of persons regularly present in the facility, both residents and staff, ^6^ IPC, i.e., infection prevention and control. ^7^ Staffing ratio, i.e., staff members per number of residents.

**Table 4:** Results of the multivariable analysis of factors associated with the outcome of a cluster vs. a sporadic case in long-term care facilities (LTCFs) in Greece during first 9 months of 2022.

Factor	OR (95% CI) ^1^	*p* Value ^2^
Pandemic phase (proxy of variants circulation)		
Peak 1	Reference	-
Plateau 1	4.57 (0.58, 43.34)	0.151
Peak 2	5.41 (0.36, 173.62)	0.258
Plateau 2	4.36 (0.23, 151.37)	0.347
Peak 3	6.80 (0.38, 234.98)	0.219
Plateau 3	- ^3^	0.988
Facility vaccination status ^4^		
Third dose > 5 months	Reference	-
Third dose ≤ 5 months	3.57 (0.40, 79.41)	0.303
Fourth dose ≤ 5 months	1.03 (0.47, 2.32)	0.942
Index case		
Staff	Reference	-
Resident	2.76 (1.44, 5.46)	0.003
Days until IPC guidance ^5^	1.38 (1.17, 1.69)	<0.001

^1^ OR-odds ratio, CI-confidence interval. Included variables are those that were statistically significant in univariate analysis and the variables under consideration (days until IPC guidance). ^2^ Statistical significance level of 5%. ^3^ Very few observations. ^4^ Vaccination status, i.e., number of LTCFs with that dose in the majority of the LTCF persons. ^5^ IPC, i.e., infection prevention and control.

**Table 5:** Results of the multivariable analysis of factors associated with attack rate (percentage of epidemiologically related COVID-19 cases in total residents and staff) in long-term care facilities in Greece during first 9 months of 2022.

Factor	Regression Coefficient	*p* Value ^1^
Pandemic phase (proxy of variants circulation)		
Peak 1	Reference	-
Plateau 1	−7.288	0.610
Peak 2	−9.473	0.562
Plateau 2	−6.536	0.713
Peak 3	−18.324	0.285
Plateau 3	−30.012	0.153
Facility vaccination status ^2^		
Third dose > 5 months	Reference	-
Third dose ≤ 5 months	3.301	0.748
Fourth dose ≤ 5 months	−5.329	0.288
Index case		
Staff	Reference	-
Resident	3.728	0.357
LTCF population size ^3^	−0.128	0.002
Staffing ratio ^4^	−7.925	0.177
Days until IPC guidance ^5^	0.621	0.118

^1^ Statistical significance level of 5%. Included variables are those that were statistically significant in univariate analysis and the variables under consideration (days until IPC guidance). ^2^ Vaccination status, i.e., number of LTCFs with that dose in the majority of the LTCF persons. ^3^ LTCF population size, i.e., total number of persons regularly present in the facility, both residents and staff. ^4^ Staffing ratio, i.e., staff members per number of residents. ^5^ IPC, i.e., infection prevention and control.

## Data Availability

The data presented in this study are available on request from the corresponding author. The data are not publicly available due to privacy reasons.
